# Screening for long noncoding RNAs associated with oral squamous cell carcinoma reveals the potentially oncogenic actions of DLEU1

**DOI:** 10.1038/s41419-018-0893-2

**Published:** 2018-08-01

**Authors:** Koyo Nishiyama, Reo Maruyama, Takeshi Niinuma, Masahiro Kai, Hiroshi Kitajima, Mutsumi Toyota, Yui Hatanaka, Tomohiro Igarashi, Jun-ichi Kobayashi, Kazuhiro Ogi, Hironari Dehari, Akihiro Miyazaki, Akira Yorozu, Eiichiro Yamamoto, Masashi Idogawa, Yasushi Sasaki, Tamotsu Sugai, Takashi Tokino, Hiroyoshi Hiratsuka, Hiromu Suzuki

**Affiliations:** 1 Department of Oral Surgery, Sapporo Medical University School of Medicine, S1, W16, Chuo-ku, Sapporo 060-8543 Japan; 2 Department of Molecular Biology, Sapporo Medical University School of Medicine, S1, W17, Chuo-ku, Sapporo 060-8556 Japan; 3 Project for Cancer Epigenomics, Cancer Institute, Japanese Foundation for Cancer Research, 3-8-31, Ariake, Koto-ku, Tokyo 135-8550 Japan; 4 Department of Medical Genome Science, Research Institute for Frontier Medicine, Sapporo Medical University School of Medicine, S1, W17, Chuo-ku, Sapporo 060-8556 Japan; 5 Department of Molecular Diagnostic Pathology, School of Medicine, Iwate Medical University, Morioka, Japan

## Abstract

Recent studies have shown that long noncoding RNAs (lncRNAs) have pivotal roles in human malignancies, although their significance in oral squamous cell carcinoma (OSCC) is not fully understood. In the present study, we identified lncRNAs functionally associated with OSCC. By analyzing RNA-seq datasets obtained from primary head and neck squamous cell carcinoma (HNSCC), we identified 15 lncRNAs aberrantly expressed in cancer tissues. We then validated their expression in 18 OSCC cell lines using qRT-PCR and identified 6 lncRNAs frequently overexpressed in OSCC. Among those, we found that knocking down DLEU1 (deleted in lymphocytic leukemia 1) strongly suppressed OSCC cell proliferation. DLEU1 knockdown also suppressed migration, invasion, and xenograft formation by OSCC cells, which is suggestive of its oncogenic functionality. Microarray analysis revealed that DLEU1 knockdown significantly affects expression of a number of cancer-related genes in OSCC cells, including HAS3, CD44, and TP63, suggesting that DLEU1 regulates HA-CD44 signaling. Expression of DLEU1 was elevated in 71% of primary OSCC tissues, and high DLEU1 expression was associated with shorter overall survival of HNSCC patients. These data suggest that elevated DLEU1 expression contributes to OSCC development, and that DLEU1 may be a useful therapeutic target in OSCC.

## Introduction

In recent years, there have been 300,000 new cases of oral cancer (2.1% of all cancers) and 145,000 deaths from the disease (2.1% of all cancers), worldwide^1^. Approximately 90% of oral cancers are histopathologically classified as squamous cell carcinoma^2^. For treatment of oral cancer, a multidisciplinary approach combining surgery, chemotherapy and radiation therapy is recommended^3^. These treatments are effective against early cancers, but are often unsatisfactory with advanced or recurrent cancers. As a result the 5-year survival rate among oral cancer patients is only about 50%^4^. In cases of inoperable or chemotherapy-resistant oral cancer, the efficacy of molecular targeted drugs, including cetuximab, a monoclonal antibody against EGFR, has been reported^5,6^. However, it is also well documented that cetuximab is less effective in cancers with *KRAS* mutations, and discovery of new therapeutic targets in oral cancer is needed^7^.

Recent genome and transcriptome analyses revealed that only 2% of the genome is translated into protein. A portion of the remaining DNA is transcribed into a large number of noncoding RNAs^8^. Long noncoding RNAs (lncRNAs) are synthesized by RNA polymerase II and share many of the biological characteristics of mRNA, though they do not encode proteins^9^. Nonetheless, evidence indicates lncRNAs have pivotal roles in human malignancies. For example, elevated expression of the lncRNA HOTAIR is strongly associated with metastasis and poor prognosis in various malignancies, including breast and gastrointestinal cancers^10,11^. In addition, HOTAIR induces epigenetic silencing of metastasis suppressor genes by recruiting Polycomb repressive complex 2 (PRC2) within cancer cells^10^. Similarly, TUG1 is overexpressed in glioma cells, where it interacts with PRC2 to suppress differentiation-associated genes^12^. Conversely, MEG3 reportedly acts as a tumor suppressor, and its expression is downregulated in various tumors, including meningioma, glioma, and gastric cancer^13^.

Dysregulation of lncRNAs has also been implicated in oral tumorigenesis. Several groups have reported that increased HOTAIR expression is associated with invasion, metastasis and stemness in oral squamous cell carcinoma (OSCC) cells^14,15^. TUG1 also reportedly promotes OSCC progression by activating Wnt/β-catenin signaling^16^. Decreased expression of MEG3 is associated with a poor prognosis in oral cancer, which consistent with the observation that MEG3 inhibits OSCC cell growth and metastasis by suppressing Wnt/β-catenin signaling^17,18^. That said, our knowledge about the role of lncRNA in oral cancer remains limited. In the present study, we aimed to identify lncRNAs that have a role in the development of OSCC. By comprehensively analyzing transcriptome datasets, we identified a series of lncRNAs overexpressed in OSCC. We then performed functional screening of the candidate lncRNAs and identified DLEU1 (deleted in lymphocytic leukemia 1) as a novel OSCC-related lncRNA. We show that elevated expression of DLEU1 likely promotes OSCC development and progression, and that DLEU1 could be a useful new therapeutic target in OSCC.

## Results

### Screening for aberrantly expressed lncRNAs in OSCC

**Fig. 1:**
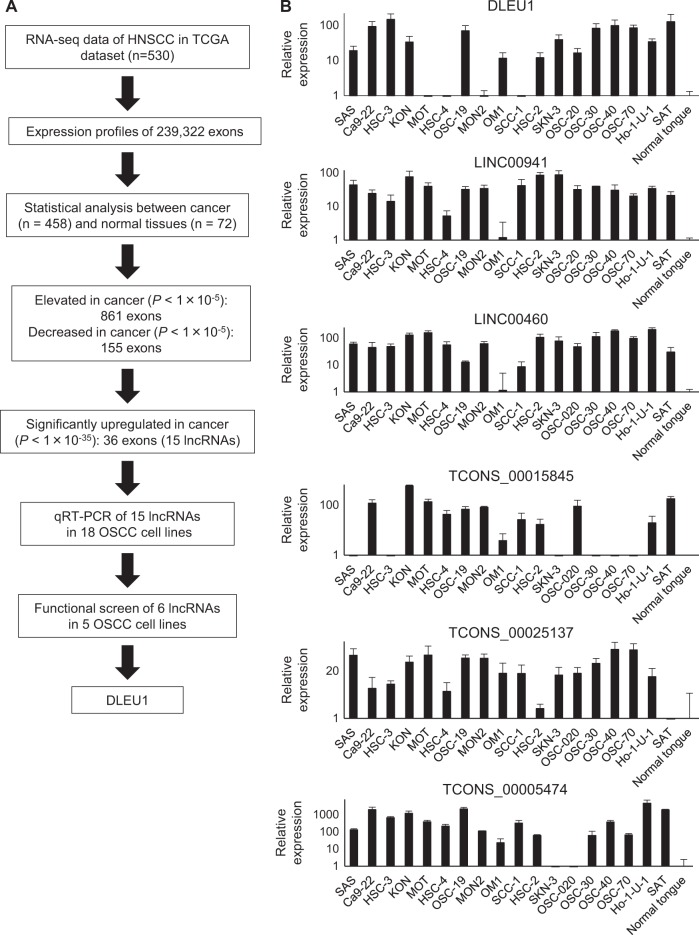
Screening for aberrantly expressed lncRNAs in OSCC. **a** Workflow of the screening to identify lncRNAs associated with OSCC. **b** Results of qRT-PCR for the 6 selected lncRNAs in 18 OSCC cell lines and a specimen of normal tongue. Results are normalized to the expression levels in normal tongue. Shown are means of three replications; error bars represent SDs

To identify lncRNAs associated with the development or progression of OSCC, we first used RNA-seq data obtained from primary HNSCC tissues in the TCGA network study (Fig. 1a). Because lncRNA genes often have multiple splice variants, we analyzed the expression levels of respective exons of the genes. We first compared the expression levels of 239,322 exons between cancer tissues (*n* = 458) and normal tissues (*n* = 72). We found that expression levels of 49,503 exons are significantly higher in cancerous than normal tissues (*P* < 1 × 10^−5^), while expression of 8724 exons is lower in cancer tissues (*P* < 1 × 10^−5^). By comparing these results with the lncRNA database, we found that the expression levels of 861 exons encoding lncRNAs are increased in cancer tissues, while levels of 155 exons are decreased (Fig. 1a)^19^. From among these, we selected 36 exons, corresponding to 15 lncRNA genes that were significantly upregulated in cancer (*P* < 1 × 10^−35^) and assessed their expression levels in a series of 18 OSCC cell lines and normal tongue tissues. qRT-PCR analysis revealed that 14 of the 15 lncRNAs were upregulated in multiple OSCC cell lines as compared to normal tissue (Supplementary Fig. 1). Expression of 6 lncRNAs (DLEU1, LINC00941, LINC00460, TCONS_00015845, TCONS_00025137, and TCONS_00005474) was significantly elevated in a majority of the OSCC cell lines, and we selected these lncRNAs for further analysis (Fig. 1b).

### Effects of lncRNA knockdown on OSCC cell proliferation

**Fig. 2:**
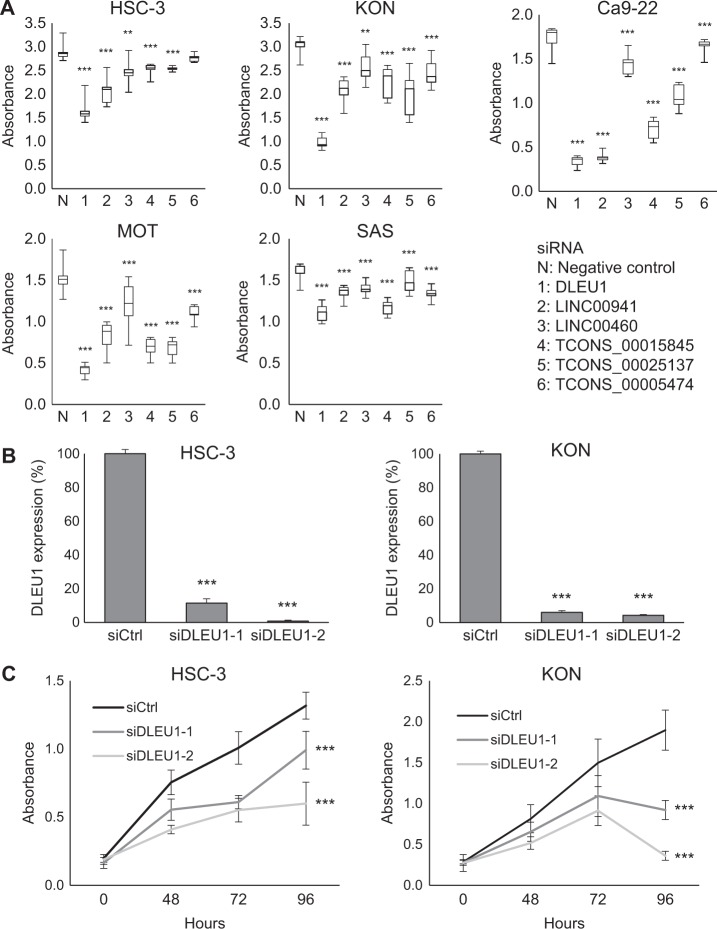
Effects of lncRNA knockdown on OSCC cell proliferation. **a** Summary of cell viability assays in the indicated OSCC cell lines with lncRNA knockdown. Cells were transfected with a siRNA targeting each lncRNA or a negative control, and cell viabilities were assessed 96 h after transfection. Shown are means of nine replications; error bars represent SDs. **b** qRT-PCR showing DLEU1 knockdown in OSCC cell lines. Cells were transfected with control siRNA (siCtrl) or siRNAs targeting DLEU1 and were harvested 72 h after transfection. **c** Cell viability assays in OSCC cell lines with DLEU1 knockdown. Cells were transfected with the indicated siRNAs, and cell viabilities were assessed at the indicated times. Shown are means of nine replications; error bars represent SDs. **P < 0.01, ***P < 0.001

To evaluate the functional importance of the identified lncRNAs, we first assessed whether knocking down expression of these lncRNAs affected OSCC cell proliferation (Fig. 2a). We found that siRNAs targeting DLEU1, LINC00941, TCONS_00015845, and TCONS_00025137 all significantly suppressed proliferation of Ca9-22 and MOT cells. DLEU1 knockdown had the strongest suppressive effect on growth in all cell lines tested. We therefore designed an additional siRNA targeting DLEU1 and confirmed that both siRNAs (siDLEU1-1 and siDLEU1-2) markedly diminished DLEU1 expression (Fig. 2b). Cell viability assays showed that knocking down DLEU1 expression with either of the two siRNAs significantly suppressed proliferation of HSC-3, KON, Ca9-22, and MOT cells (Fig. 2c, Supplementary Fig. 2). Notably, DLEU1 knockdown not only suppressed KON cell proliferation, it also significantly decreased cell viability 96 h after transfection. This suggests DLEU1 knockdown induced KON cell death (Fig. 2c).

### Oncogenic function of DLEU1 in OSCC cells

**Fig. 3:**
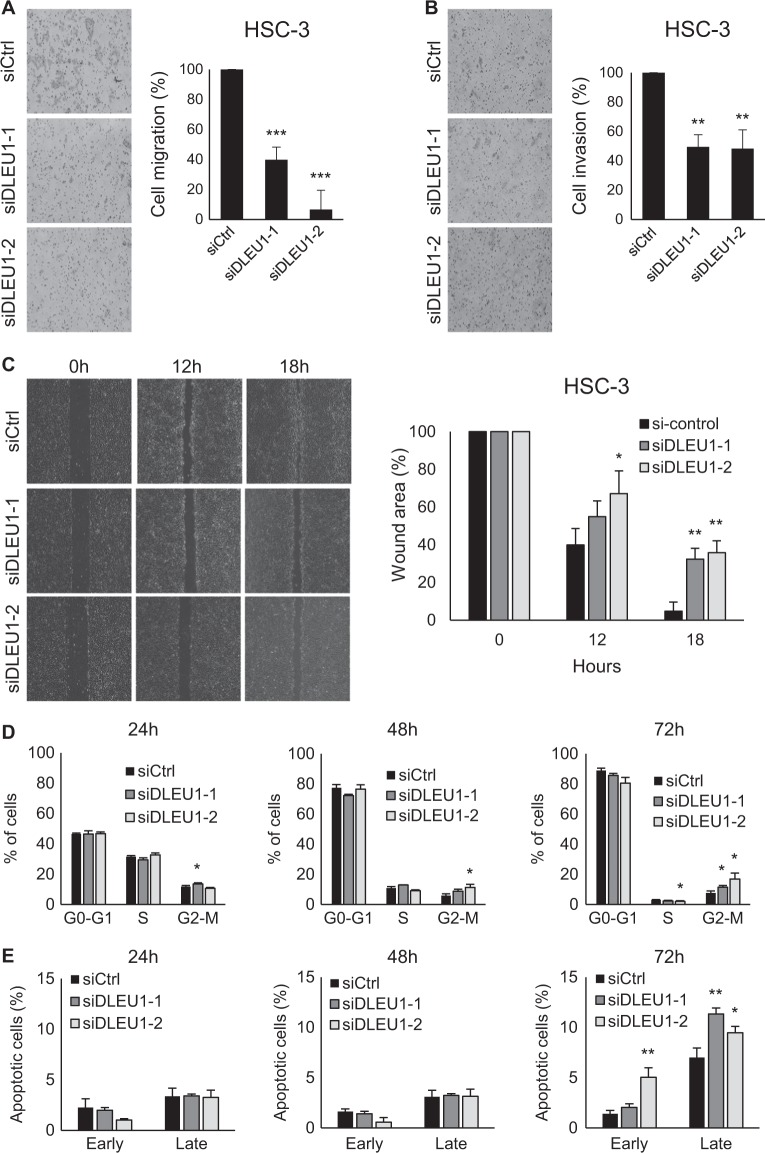
Functional analysis of DLEU1 in OSCC cells. **a**, **b** Results of migration (**a**) and invasion (**b**) assays with HSC-3 cells transfected with siRNAs targeting DLEU1 or control siRNA (siCtrl). Representative results are on the left, and summarized results are on the right. Shown are means of five random microscopic fields per membrane; error bars represent SDs. **c** Results of wound healing assays using HSC-3 cells transfected with the indicated siRNAs. Representative results are on the left, and summarized results are on the right. Shown are means of three replications; error bars represent SDs. **d**, **e** Results of cell cycle (**d**) and apoptosis (**e**) analyses in HSC-3 cells. Cells were transfected with the indicated siRNAs, after which cell cycle and apoptosis were assessed at the indicated time points. Summarized results of three replications are shown; error bars represent SDs. *P < 0.05, **P < 0.01, ***P < 0.001

We next tested whether DLEU1 is involved in migration and invasion by OSCC cells. Transwell chamber assays revealed that DLEU1 knockdown suppresses migration of HSC-3, KON, and Ca9-22 cells (Fig. 3a, Supplementary Fig. 3). Matrigel invasion assays showed that DLEU1 knockdown suppresses invasion by OSCC cells (Fig. 3b, Supplementary Fig. 3). The inhibitory effect of DLEU1 knockdown on cell migration was confirmed in wound healing assays (Fig. 3c, Supplementary Fig. 4).

When we then assessed the involvement of DLEU1 in cell cycle and apoptosis, we found that DLEU1 knockdown induces G2/M arrest in OSCC cells (Fig. 3d, Supplementary Figs. 5, 6). DLEU1 knockdown also upregulated expression of a G2 checkpoint kinase, WEE1, in OSCC cells, which is consistent with the cell cycle assay results (Supplementary Fig. 7). In addition, there was a mild increase in the G1 phase population and reduction in the S phase population of KON and Ca9-22 cells, which suggests DLEU1 depletion induces G1/S arrest (Supplementary Figs. 5, 6). DLEU1 knockdown also induced apoptosis in OSCC cells (Fig. 3e, Supplementary Figs. 8 and 9). The incidence of apoptosis was greater in KON than HSC-3 cells, which is consistent with the results of the cell viability assays (Fig. 2, Supplementary Fig. 8). These results suggest that DLEU1 contributes to OSCC progression by promoting proliferation, migration, and invasion by OSCC cells and by inhibiting apoptosis.

**Fig. 4:**
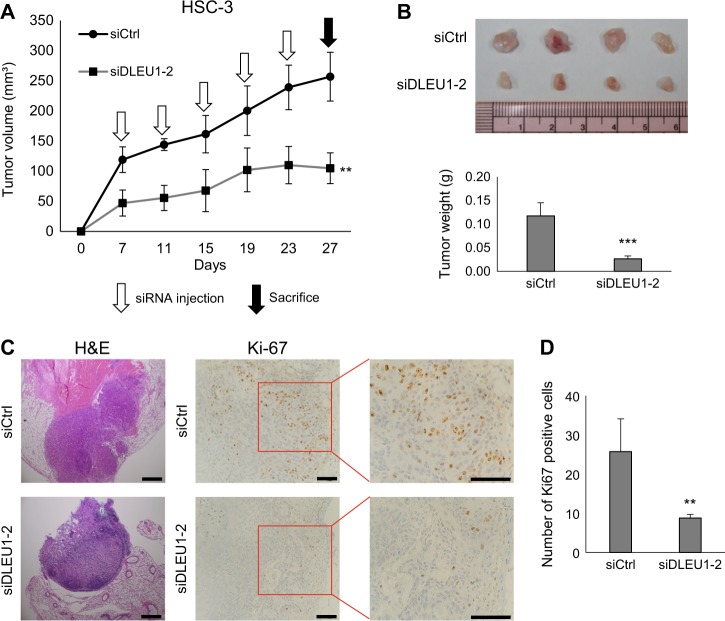
Effects of DLEU1 knockdown on xenograft tumor formation by OSCC cells in mice. **a** Tumor growth in nude mice injected with HSC-3 cells transfected with siRNA targeting DLEU1 or control siRNA (siCtrl). Intratumoral injection of siRNAs was also performed at the indicated times. Shown are means of four replications; error bars represent SDs. **b** Resected tumors (upper) and tumor weights (lower) in the indicated groups. Shown are means of four replications; error bars represent SDs. **c** Representative views of hematoxylin and eosin (left) and Ki-67 (right) staining in tumors treated with the indicated siRNAs. Scale bars, 200 µm. **d** Summary of Ki-67-positive cell counts. Shown are means of four replications; error bars represent SDs. **P < 0.01, ***P < 0.001

To confirm the oncogenic functionality of DLEU1 in vivo, we performed mouse xenograft experiments, which showed that siRNA targeting DLEU1 significantly suppressed xenograft tumor formation by OSCC cells in nude mice (Fig. 4a, b). Immunohistochemical analysis revealed that Ki-67 expression was decreased in tumors treated with the DLEU1 siRNA, which indicates DLEU1 knockdown suppresses tumor cell proliferation (Fig. 4c, d).

### Effects of DLEU1 knockdown on gene expression profiles in OSCC cells

**Fig. 5:**
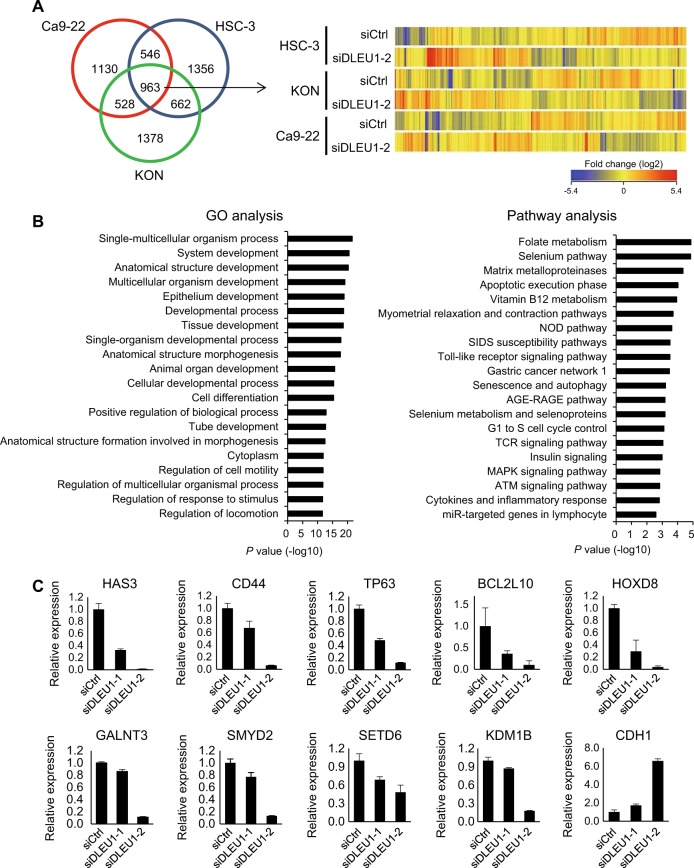
Effects of DLEU1 knockdown on gene expression profiles in OSCC cells. **a** OSCC cell lines were transfected with siRNA targeting DLEU1 (siDLEU1-2) or control siRNA (siCtrl), after which microarray analysis was performed. A Venn diagram showing genes whose expression was altered by DLEU1 knockdown (>2-fold) in the indicated OSCC cell lines is on the left. A heatmap for the expression of 963 selected probe sets is on the right. **b** Results of GO (left) and pathway analyses (right) of the 963 probes. **c** qRT-PCR for cancer-related genes in HSC-3 cells transfected with the indicated siRNAs. Shown are means of three replications; error bars represent SDs

To further clarify the molecular function of DLEU1 in OSCC, we performed a gene expression microarray analysis with three OSCC cell lines (HSC-3, KON, and Ca9-22) transiently transfected with siRNA targeting DLEU1 (siDLEU1-2) or control siRNA. We found that expression of 963 probe sets (814 unique genes) were altered (>2-fold) by DLEU1 knockdown in all three cell lines tested (Fig. 5a, Supplementary Table 1). Gene ontology (GO) analysis showed that genes associated with “development”, “differentiation” and “regulation of cell motility” were enriched among the affected genes (Fig. 5b). Pathway analysis also revealed that genes associated with “matrix metalloproteinases”, “apoptosis”, “G1 to S cell cycle”, and “MAPK signaling” were enriched among the affected genes (Fig. 5b). qRT-PCR performed to validate the microarray results showed that expression of a number of cancer-related genes, including HAS3, CD44, TP63, and SMYD2, was decreased by siRNAs targeting DLEU1, while expression of CDH1 was increased by DLEU1 knockdown. Western blot analysis of HAS3, CD44, TP63, and SMYD2 gave results similar to those obtained with qRT-PCR (Supplementary Fig. 10). This suggests that these genes may be downstream targets of DLEU1 in OSCC cells (Fig. 5c).

### Elevated expression of DLEU1 in primary OSCC tissues

**Fig. 6:**
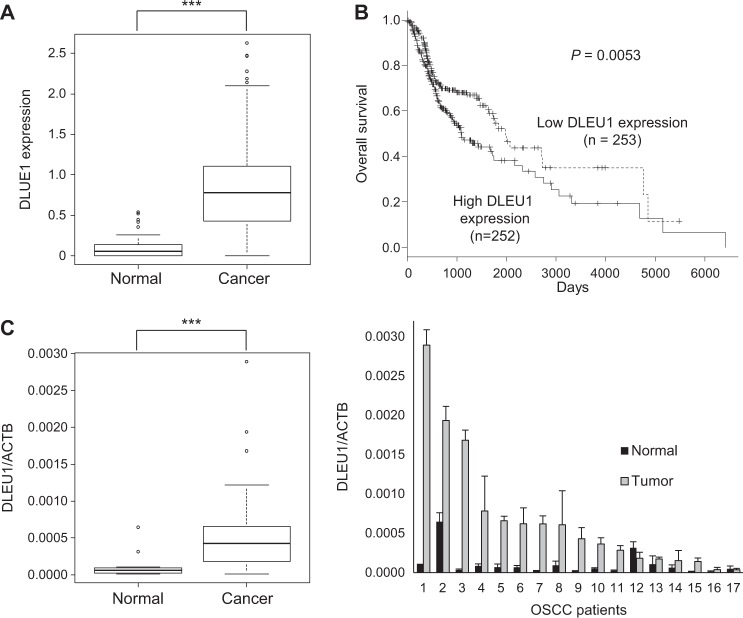
Elevated expression of DLEU1 in primary tumors. **a** Summaries of DLEU1 expression in normal tissue and primary HNSCC tumors in TCGA datasets. **b** Kaplan–Meier curve showing the effect of DLEU1 expression on survival of HNSCC patients (*n* = 505). **c** Summarized qRT-PCR results for DLEU1 in primary tumors (*n* = 29) and adjacent normal tissues (*n* = 17) obtained from Japanese OSCC patients. **d** qRT-PCR for DLEU1 in paired normal tissue and primary tumors. ***P < 0.001

We investigated the clinical relevance of DLEU1 expression in OSCC using RNA-seq data obtained from primary HNSCC in The Cancer Genome Atlas (TCGA) study. We found that expression of DLEU1 is significantly higher in cancerous (*n* = 505) than normal (*n* = 72) tissues (Fig. 6a). Kaplan–Meier curves revealed that higher levels of DLEU1 expression are associated with poorer overall survival among HNSCC patients (Fig. 6b). Then in a series of 29 samples of primary OSCC tissue and 17 adjacent normal tissue samples obtained from Japanese patients, we observed that DLEU1 expression is significantly elevated in cancer tissues (Fig. 6c). Moreover, by comparing the 17 pairs of OSCC tissue with matched adjacent normal tissues, we found that DLEU1 expression was higher in 12 (71%) tumors than in their normal counterparts (Fig. 6d). Finally, comparison of DLEU1 expression levels with those of putative target genes in primary HNSCC in TCGA datasets revealed that DLEU1 expression positively correlates with HAS3, CD44, TP63, BCL2L10, SMYD2, KDM1B, and GALNT3 expression, which suggests expression of these genes may be regulated by DLEU1 in primary tumors (Supplementary Fig. 11).

## Discussion

In the present study, we identified a series of lncRNAs aberrantly expressed in OSCC. Although the expression levels of lncRNA genes are generally lower than those of protein-coding genes, we found that a subset of lncRNAs are significantly and frequently overexpressed in OSCC as compared to normal tissues. The list of genes we identified in this study includes multiple lncRNAs reportedly implicated in human malignancies. For instance, LINC00460 is upregulated in HNSCC and kidney cancer, and its elevated expression is associated with poorer survival^20^. Another study reported that elevated expression of LINC00460 is associated with advanced clinical stages, lymph node metastasis, and a poor prognosis in esophageal squamous cell carcinoma (ESCC), and that LINC00460 knockdown induces cell cycle arrest and apoptosis in ESCC cells^21^. By contrast, higher expression of LINC00941 is reportedly associated with better survival of lung adenocarcinoma patients, although its function remains unclear^22^. Thus, the lncRNAs we identified in this study may be associated with the biological and clinical characteristics of OSCC.

Among the candidate lncRNAs examined, we found that knocking down DLEU1 had the strongest anti-proliferative effects in multiple OSCC cell lines. DLEU1 is located at 13q14.3, a region recurrently deleted in B-cell chronic lymphocytic leukemia (B-CLL)^23,24^. Because 13q14 is a tumor suppressor locus that contains RB1, DLEU1 is thought to be a putative tumor suppressor gene^23^. However, DLEU1 shows limited homology to known genes, and no mutations of DLEU1 have been found in B-CLL^23,25^. Sequencing analysis of RNA variants suggests DLEU1 encodes a noncoding RNA, but the tumor suppressive function of DLEU1 is not fully understood^26^. A recent study showed that expression of DLEU1 is upregulated via DNA demethylation in CLL, and that epigenetic aberration is associated with downregulation of neighboring tumor suppressor genes that regulate NF-κB signaling^27^.

Recent studies also suggest DLEU1 has a role in other human malignancies. In Burkitt lymphoma cells, DLEU1 knockdown significantly inhibits apoptosis and promotes cell proliferation, suggesting it acts as a tumor suppressor^28^. By contrast, DLEU1 is overexpressed in epithelial ovarian carcinoma (EOC), where it contributes to tumor development through direct interaction with miR-490-3p^29^. miR-490-3p acts as a tumor suppressor by targeting CDK1 in EOC, and DLEU1 may promote tumorigenesis by inhibiting miR-490-3p function^29^.

Because multiple variants are transcribed from the DLEU1 gene, we used siRNAs that targeted all three variants annotated in the RefSeq database (NR_109973, NR_002605, and NR_109974). We found that DLEU1 knockdown inhibited proliferation, migration, and invasion by OSCC cells. Depletion of DLEU1 induced cell cycle arrest and apoptosis in OSCC cells, which could have an anti-proliferative effect. We also found that administration of siRNA targeting DLEU1 suppressed tumor formation by OSCC cells in vivo. Elevated expression of DLEU1 was frequently observed in clinical OSCC tumors, and high DLEU1 expression was associated with poorer survival among HNSCC patients. These results suggest that upregulation of DLEU1 may promote OSCC development and progression, and that DLEU1 is a potential therapeutic target in OSCC.

To further clarify the molecular function of DLEU1 in OSCC, we examined the effect of DLEU1 knockdown on gene expression profiles in OSCC. Microarray analysis revealed that DLEU1 may regulate expression of a number of cancer-related genes. For instance, DLEU1 knockdown led to decreased expression of HAS3 and CD44. HAS3 (hyaluronan synthase 3) is also strongly implicated in cancer development. Hyaluronan (HA) is an extracellular, cell-surface-associated polysaccharide that regulates cell adhesion, migration, and proliferation^30^, and overexpression of its synthetic enzyme, HAS3, is reportedly associated with cancer cell growth and metastasis^31,32^. A recent study also showed that HAS3 exerts an oncogenic effect in oral cancer through formation of an inter-regulatory loop with TNF-α^33^. CD44 is a transmembrane protein known to be a cancer stem cell marker in HNSCC^34^. CD44 is a major HA receptor, and interaction between HA and CD44 promotes HNSCC progression and chemoresistance through activation of EGFR signaling^35^. Moreover, a recent study showed that ΔNp63, which is an isoform of p63 and is overexpressed in a majority of HNSCCs, mediates HA metabolism though direct regulation of HAS3 and CD44 expression in HNSCC^36^. In that context, our results suggest that *DLEU1* may contribute to OSCC development through interaction with HA-CD44 signaling.

We also noted that DLEU1 knockdown suppressed expression of genes encoding the histone methylation modifiers SMYD2, SETD6, and KDM1B. SMYD2 was identified as a lysine methyltransferase (KMT) for histone H3K36 and K370 of p53, and it is reportedly overexpressed in various tumors, including HNSCC^37,38^. Recent studies also show that SMYD2 mediates methylation of proteins critical for oncogenesis, including β-catenin and EML4-ALK^39,40^. SETD6 was first identified as a KMT for histone H2AZ that controls expression of estrogen-responsive genes and proliferation in breast cancer cells^41^. Another study reported that SETD6 is overexpressed in bladder cancer, where it stimulates NF-κB signaling by mediating methylation of p65^42^. KDM1B (also known as LSD2) is a histone H3K4 demethylase reportedly involved in regulating DNA methylation and proliferation in breast cancer cells^43,44^.

In contrast to genes described above, we found that DLEU1 knockdown induces the expression of CDH1 in OSCC cells. CDH1 encodes the cell adhesion molecule E-cadherin, which is strongly implicated in invasion and metastasis in cancers of epithelial origin. CDH1 is frequently silenced in OSCC cells by CpG island hypermethylation, and CDH1 methylation and reduced E-cadherin expression are associated with invasion and metastasis in OSCC^45–47^. These results suggest that altered expression of multiple cancer-related genes by DLEU1 knockdown contributes to the antitumor effects seen in OSCC cells. However, the specific molecular mechanism by which DLEU1 regulates expression of target genes remains unknown. Earlier studies have reported that lncRNAs regulate transcription through chromatin modulation, but more recent studies showed that lncRNAs modulate more diverse cellular functions though interaction with a variety of molecules^48^. Thus, further study will be necessary to clarify the function of DLEU1 in OSCC.

In summary, we show that expression of a substantial number of lncRNAs are dysregulated in OSCC. Elevated expression of DLEU1 may be causally associated with oral carcinogenesis, and DLEU1 may be a prognostic marker and therapeutic target in OSCC. We anticipate that further study of lncRNA regulation and its functional significance in OSCC will provide new strategies for the treatment of OSCC patients.

## Materials and methods

### Cell lines and tissue samples

OSCC cell lines were obtained and cultured as described previously^49^. Primary OSCC tissues were collected from Japanese patients who underwent surgical resection at the Department of Oral Surgery, Sapporo Medical University between 2015 and 2017. A series of 29 samples of primary OSCC tissue and 17 samples of adjacent non-tumorous tissue were obtained through surgical resection or biopsy. Informed consent was obtained from all patients before collection of the specimens. Approval for this study was obtained from the Institutional Review Board of Sapporo Medical University. Total RNA was extracted using an RNeasy Mini kit (Qiagen, Hilden, Germany). Total RNA from normal tongue tissue from a healthy individual was purchased from BioChain (Hayward, CA, USA).

### Quantitative reverse-transcription PCR

Single-stranded cDNA was prepared using PrimeScript RT Master Mix Perfect Real Time (Takara Bio Inc, Kusatsu, Japan). Quantitative reverse-transcription PCR (qRT-PCR) was performed using SYBR Select Master Mix (Thermo Fisher Scientific, Waltham, MA, USA) and a model 7500 Fast Real-Time PCR System (Thermo Fisher Scientific). ACTB (β-actin) was used as an endogenous control for qRT-PCR. Primer sequences are listed in Supplementary Table 2.

### RNA interference

For RNA interference-induced knockdown of lncRNAs, OSCC cells (5 × 10^3^ cells per well in 96-well plates or 1 × 10^6^ cells per well in 6-well plates) were transfected with 2.2 pmol (96-well) or 44 pmol (6-well) of siRNAs targeting lncRNAs (Sigma-Aldrich, St Louis, MO, USA) or Mission siRNA Universal Negative Control #1 (Sigma-Aldrich) using Lipofectamine RNAiMAX (Thermo Fisher Scientific). We tested multiple doses of siRNAs and used the lowest dose that provided a sufficient knockdown effect. Sequences of the siRNAs are listed in Supplementary Table 3.

### Cell viability assay

OSCC cells (5 × 10^3^ cells per well in 96-well plates) were transfected with siRNAs as described above. To assess the effects of lncRNAs, cell viability assays were carried out 96 h after transfection using a Cell Counting kit-8 (Dojindo, Kumamoto, Japan) according to the manufacturer’s instructions. To assess the effect of DLEU1 knockdown, cell viability assays were carried out 48, 72, and 96 h after transfection.

### Cell migration and invasion assays

Cells were transfected with siRNAs as described above. Transwell chambers were used for cell migration (BioCoat Control Insert 24-well plate 8.0 μm; Corning Inc., Corning, NY, USA) and invasion (BioCoat Matrigel Invasion Chamber 24-well plate 8.0 μm; Corning Inc.) analyses. Cells were harvested 24 h after transfection and resuspended in serum-free RPMI 1640 medium, after which 5 × 10^4^ cells were added to the upper chamber. RPMI 1640 medium with 10% fetal bovine serum was added to the lower well. After incubation for 24 h at 37 °C, migrating or invading cells on the lower surface of the filter were fixed and stained using a Diff-Quik staining kit (Sysmex, Tokyo, Japan). Cell numbers were determined by counting in five randomly selected microscope fields per membrane.

### Wound healing assay

OSCC cells (1 × 10^6^ cells per well in 6-well plates) were transfected with siRNAs as described above. Twenty-four hours after transfection, linear scars were drawn in the monolayer using a pipette tip, and the cells were incubated for an additional 18 h. Photomicrographs of the scars and invading cells then taken to evaluate wound closure. The experiments were performed in triplicate for each condition.

### Cell cycle and apoptosis assays

Cells were transfected with siRNAs and incubated for 24, 48, or 72 h, after which they were treated using a Click-iT Plus EdU Alexa Fluor 647 Flow Cytometry Assay Kit (Thermo Fisher Scientific) or ApoScreen Annexin V Apoptosis Kit (Southern Biotech, Birmingham, AL, USA) according to the manufacturers’ instructions. Flow cytometric analyses were performed using a BD FACSCanto II (BD Biosciences, Franklin Lakes, NJ, USA) with BD FACSDiva software (BD Biosciences). Data analysis was performed using FlowJo software (FlowJo, LLC, Ashland, OR, USA). Apoptosis was also assessed by detecting caspase-3 activity using an APOPCYTO Caspase-3 Fluorometric Assay Kit (MBL, Nagoya, Japan) and an Infinite M1000 PRO (TECAN, Männedorf, Switzerland) according to manufactures’ instructions.

### Xenograft studies

All animal studies were performed in accordance with the protocols approved by the institutional animal ethical committee at Sapporo Medical University. Mixtures of 1 × 10^7^ siRNA-transfected HSC-3 cells plus 0.2 ml of Matrigel basement matrix (Corning Inc. Corning, NY, USA) were injected subcutaneously in the bilateral thighs of 6-week-old female BALB/cAJcl-nu mice. In vivo siRNA transfection was then performed using AteloGene Local Use Quick Gelation (Koken Co, Tokyo, Japan) according to manufacturer’s instructions. Beginning 1 week after tumor cell inoculation, intratumoral injection of siRNA (250 pmol) with atelocollagen mixtures (50 μl) was administered every 4 days. Tumor size was measured every 4 days using digital calipers, and tumor volume was calculated using the formula length × width^2^/2. Tumor tissues were immunohistochemically stained using a mouse anti-Ki67 monoclonal antibody (MIB-1, 1:100 dilution, M7240, Dako, Glostrup, Denmark). Numbers of Ki-67-positive cells were determined by counting in five randomly selected microscope fields per tumor.

### Gene expression microarray analysis

Cells were transfected with siRNAs as described above, and total RNA was extracted 72 h after transfection. Gene expression microarray analysis was carried out according to the manufacturer’s instructions (Agilent Technologies, Santa Clara, CA, USA). Briefly, 100 ng of total RNA were amplified and labeled using a Low-input Quick Amp Labeling kit One-color (Agilent Technologies), after which the synthesized cRNA was hybridized to a SurePrint G3 Human GE microarray v2 (G4851; Agilent Technologies). The microarray data were analyzed using GeneSpring GX version 13 (Agilent Technologies). The Gene Expression Omnibus accession number for the microarray data is GSE108851.

### Western blot analysis

Seventy-two hours after transfecting cells with siRNAs as described above, the cellular proteins were extracted and western blot analysis was performed as described previously^50^. Mouse anti-CD44 monoclonal antibody (1:1000 dilution, clone 8E2, Catalog No 5640, Cell Signaling Technology, Danvers, MA, USA), mouse anti-HAS3 monoclonal antibody (1:1000 dilution, clone 3C9, Catalog No MA5-17088, Thermo Fisher Scientific), rabbit anti-p63 polyclonal antibody (1:1000 dilution, clone N2C1, Catalog No GT102425, Gene Tex, Irvine, CA, USA), rabbit anti-SMYD2 monoclonal antibody (1:1000 dilution, clone D14H7, Catalog No 9734, Cell Signaling Technology), and mouse anti-β-actin monoclonal antibody (1:2000 dilution, clone AC-15, Catalog No A5441, Sigma-Aldrich) were used.

### Data analysis

RNA sequencing (RNA-seq) data from head and neck squamous cell carcinoma (HNSCC) in The Cancer Genome Atlas (TCGA) datasets were obtained from the UCSC Cancer Genomics Browser (https://genome-cancer.ucsc.edu). Quantitative variables were analyzed using Student’s *t*-test. Survival was analyzed using the log-rank test for two group comparisons. Values of *P* < 0.05 (two-sided) were considered statistically significant. Statistical analyses were carried out using EZR version 1.32 (Saitama Medical Center, Jichi Medical University, Saitama, Japan)^51^.

## Electronic supplementary material

Supplementary Figures

Supplementary Table 1

Supplementary Table 2

Supplementary Table 3
