# Supplementary material for: Screening for long noncoding RNAs associated with oral squamous cell carcinoma reveals the potentially oncogenic actions of DLEU1
Source: Cell Death Dis. 2018 Aug 1;9(8):826. doi: 10.1038/s41419-018-0893-2 (PMC6070574; doi:10.1038/s41419-018-0893-2)
Supplement: Supplementary file 1 — Supplementary Figures [file 41419_2018_893_MOESM1_ESM.doc]

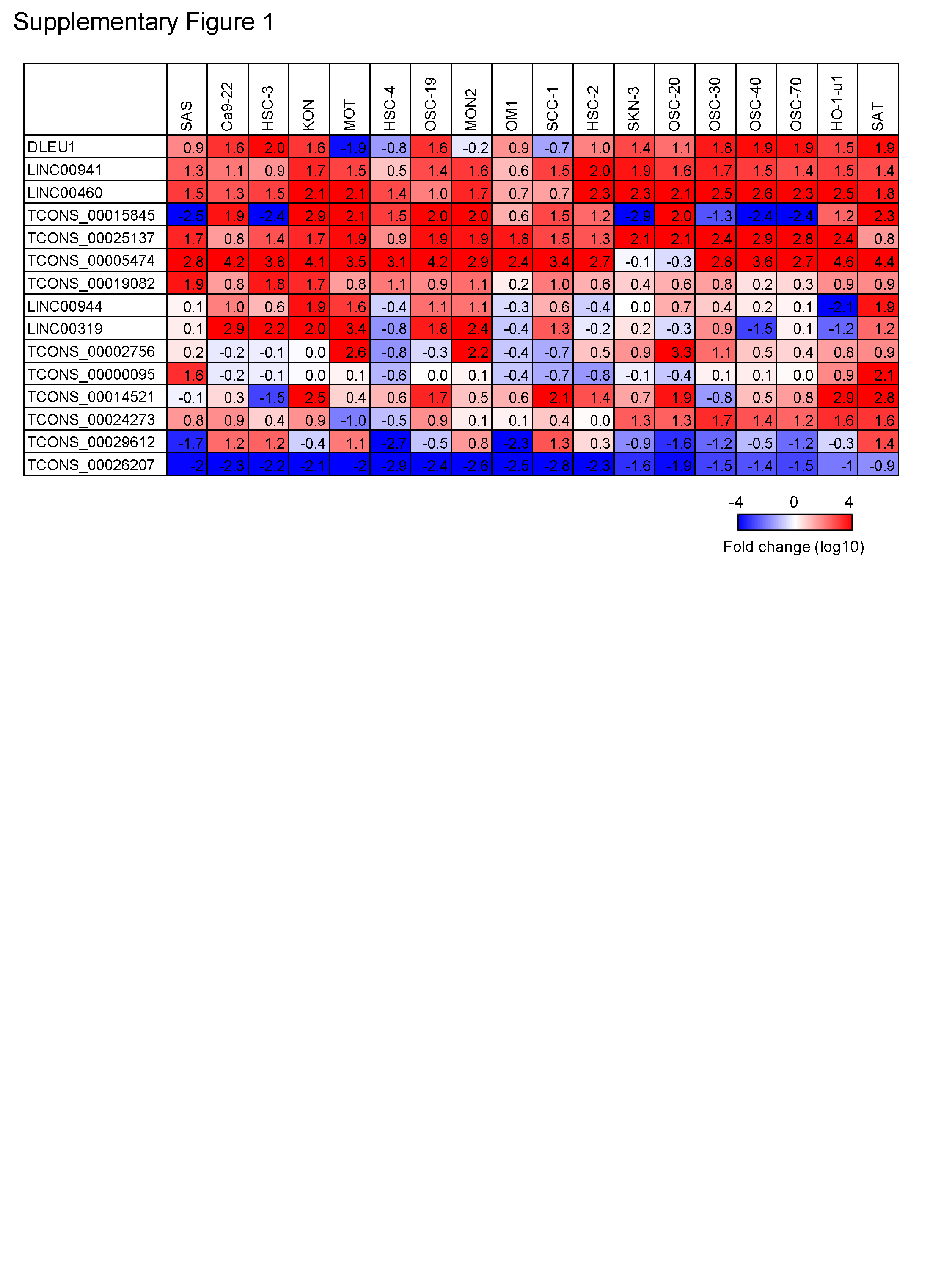


**Supplementary Figure 1**. Summarized results of qRT-PCR for 15 lncRNAs in 18 OSCC cell lines. Results are normalized to the expression levels in normal tongue tissue.


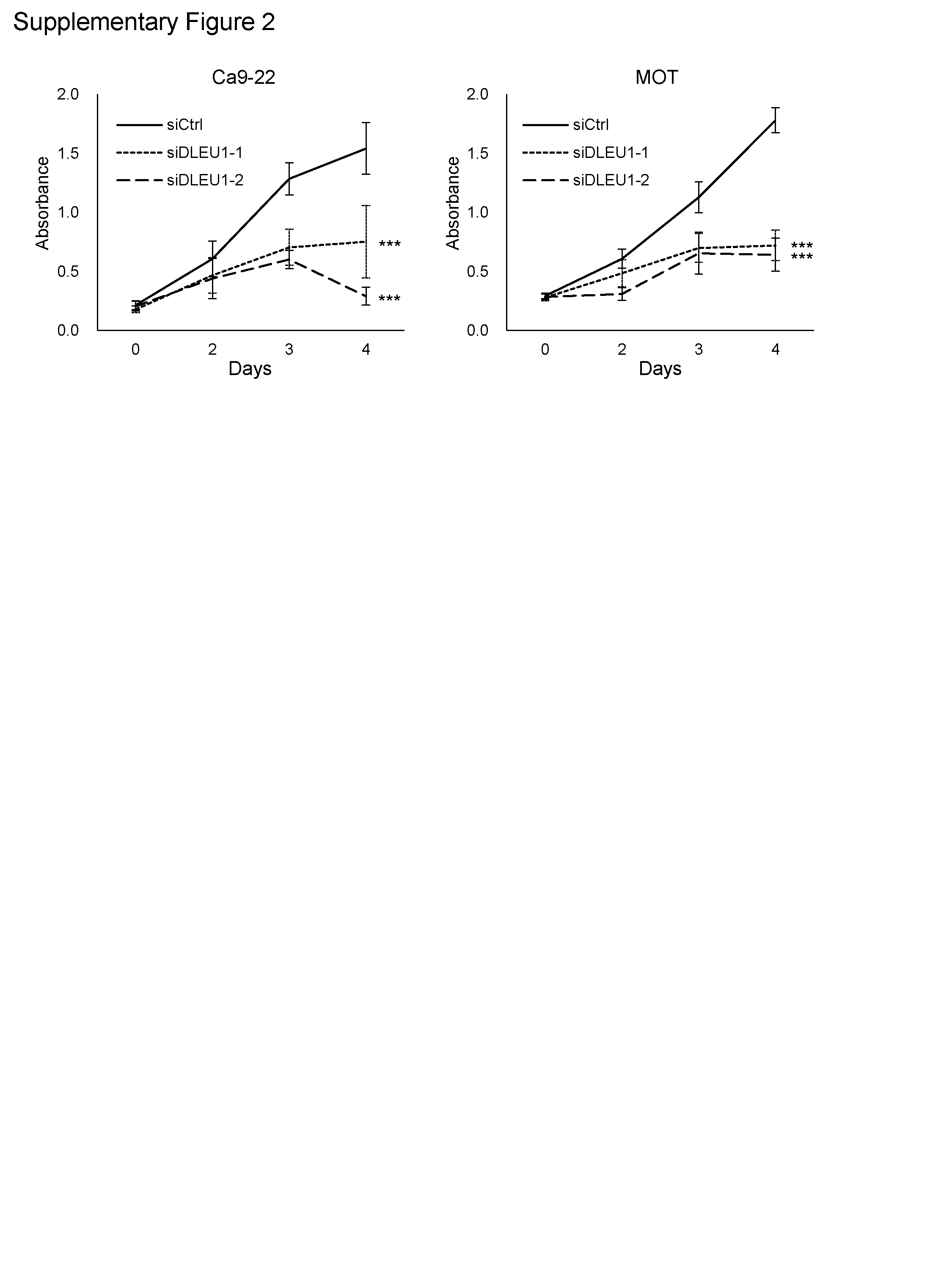


**Supplementary Figure 2**. Cell viability assays in OSCC cell lines with DLEU1 knockdown. Cells were transfected with siRNAs targeting DLEU1 (siDLEU1-1 and siDLEU1-2) or control siRNA (siCtrl), and cell viabilities were assessed at the indicated times. Shown are means of 9 replications; error bars represent SDs. ****P* < 0.001.


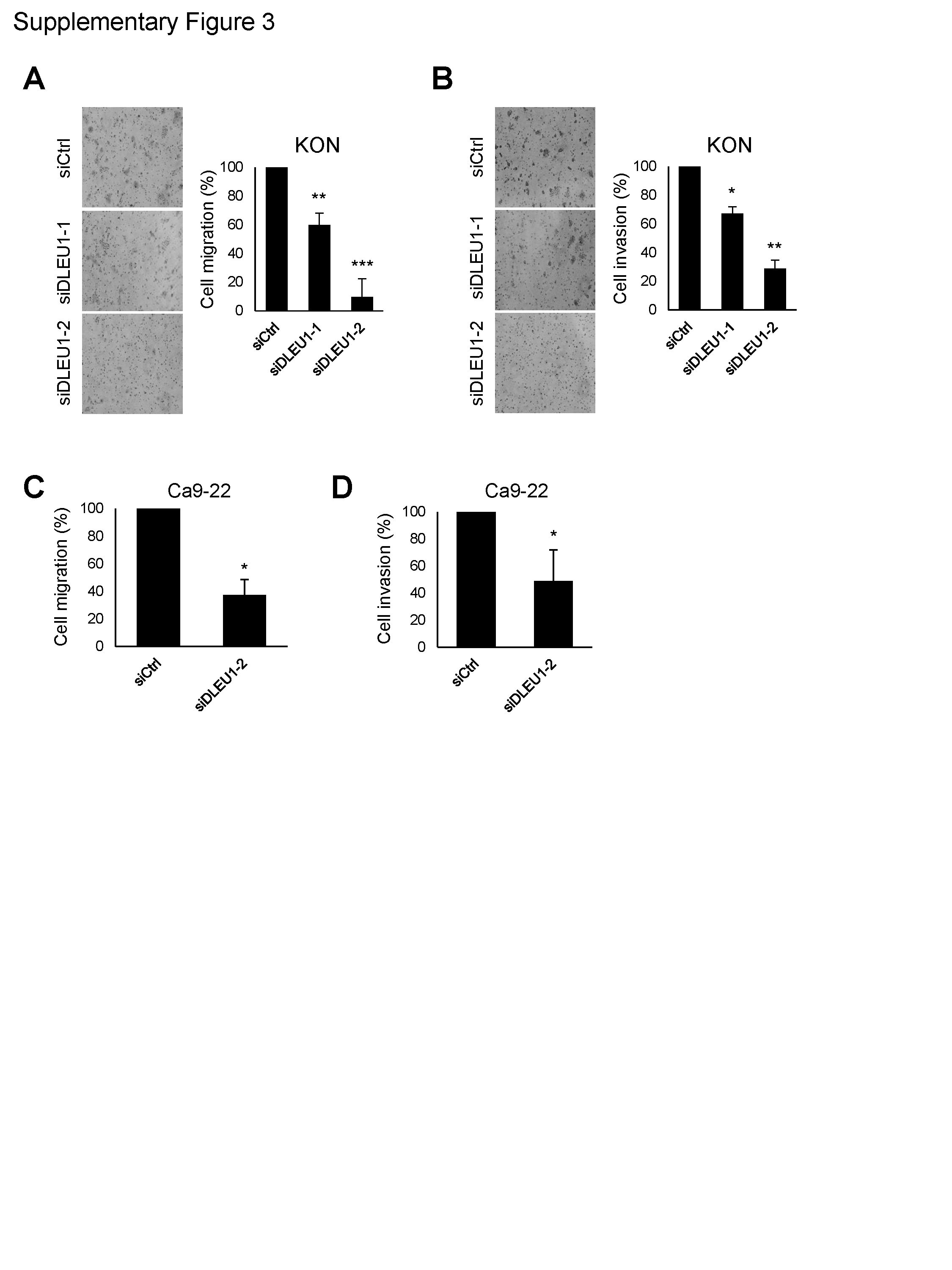


**Supplementary Figure 3**. Effects of DLEU1 knockdown on OSCC cell migration and invasion. (A,B) Results of migration (A) and invasion (B) assays with KON cells transfected with siRNAs targeting DLEU1 or control siRNA (siCtrl). Representative results are on the left, and summarized results are on the right. Shown are means of 5 random microscopic fields per membrane; error bars represent SDs. (C,D) Results of migration (C) and invasion (D) assays with Ca9-22 cells transfected with the indicated siRNAs. Shown are means of 5 random microscopic fields per membrane; error bars represent SDs. **P* < 0.05, ***P* < 0.01, ****P* < 0.001.


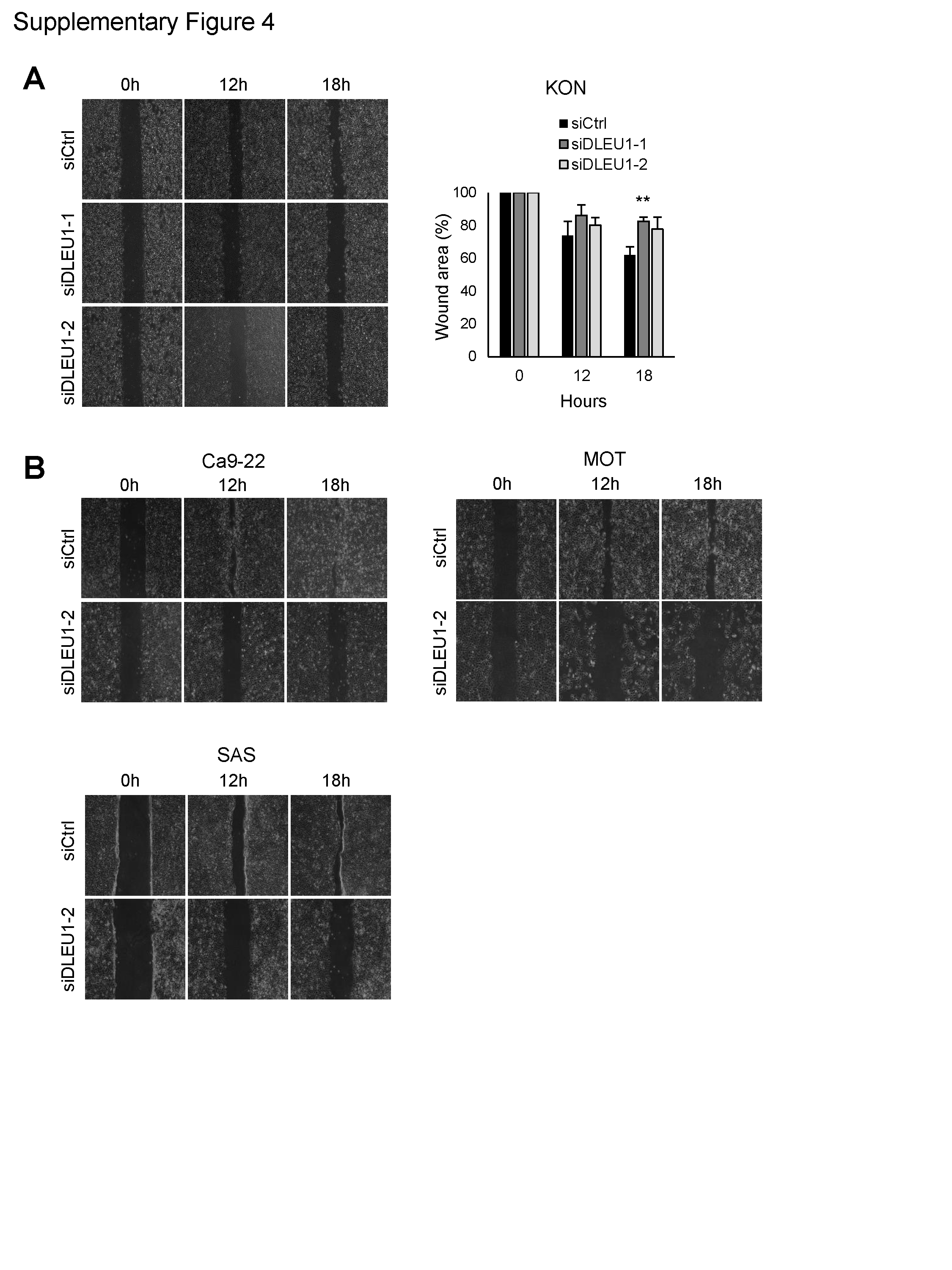


**Supplementary Figure 4**. Effects of DLEU1 knockdown on OSCC cell migration. (A) Results of wound healing assays using KON cells transfected with siRNAs targeting DLEU1 or control siRNA (siCtrl). Representative results are on the left, and summarized results are on the right. Shown are means of 3 replications; error bars represent SDs. ***P* < 0.01. (B) Wound healing assays using the indicated OSCC cell lines transfected with the indicated siRNAs.


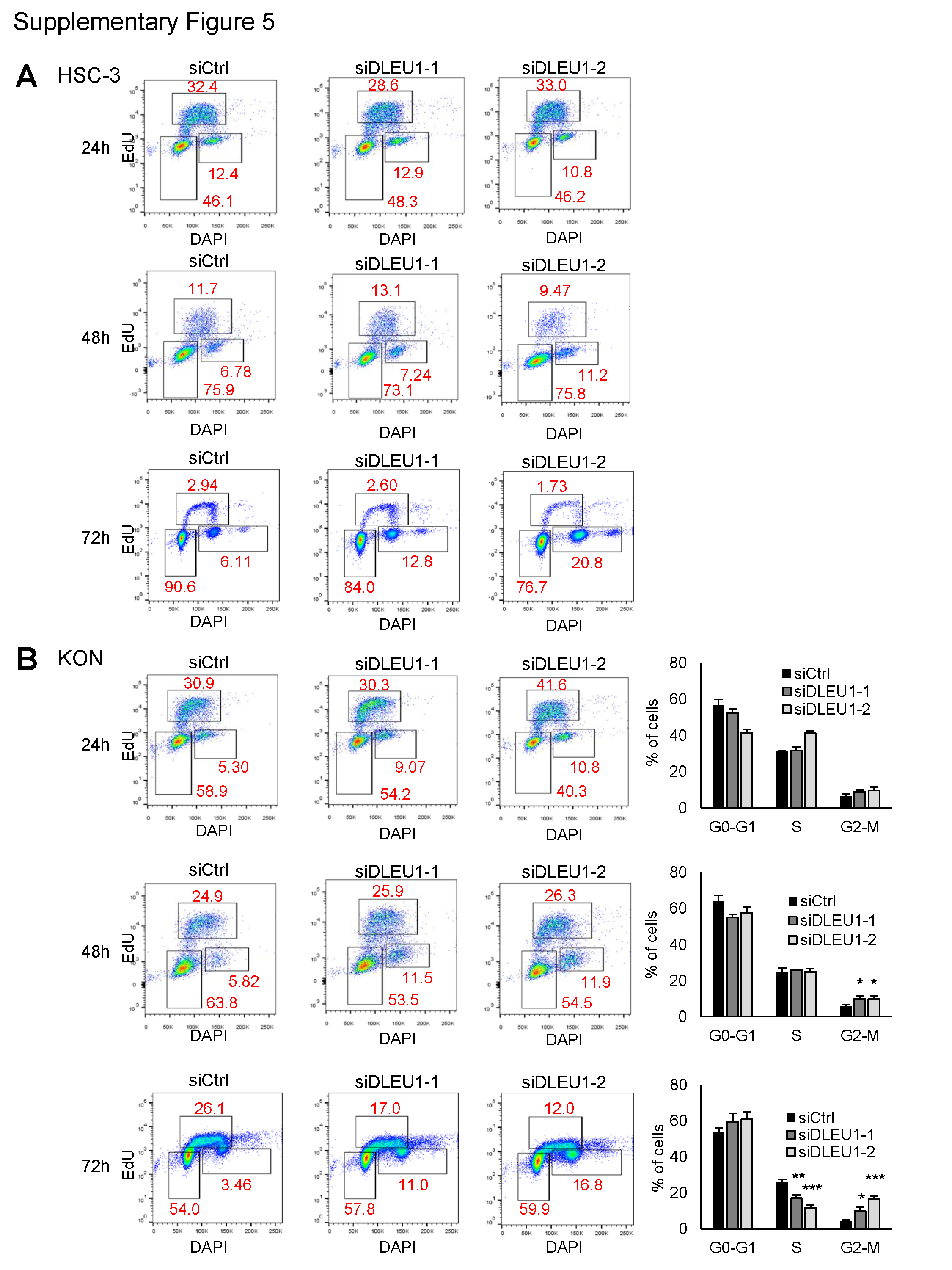


**Supplementary Figure 5**. Effects of DLEU1 knockdown on cell cycle in OSCC cells. (A) Representative results of cell cycle analyses of HSC-3 cells transfected with siRNAs targeting DLEU1 or control siRNA (siCtrl). Cells were transfected with the indicated siRNAs, and cell cycling was assessed at the indicated time points. Summarized results are shown in Figure 3. (B) Results of cell cycle analyses of KON cells transfected with the indicated siRNAs. Representative results are shown on the left. Summarized results of 3 replications are on the right; error bars represent SDs. **P* < 0.05, ***P* < 0.01, ****P* < 0.001.


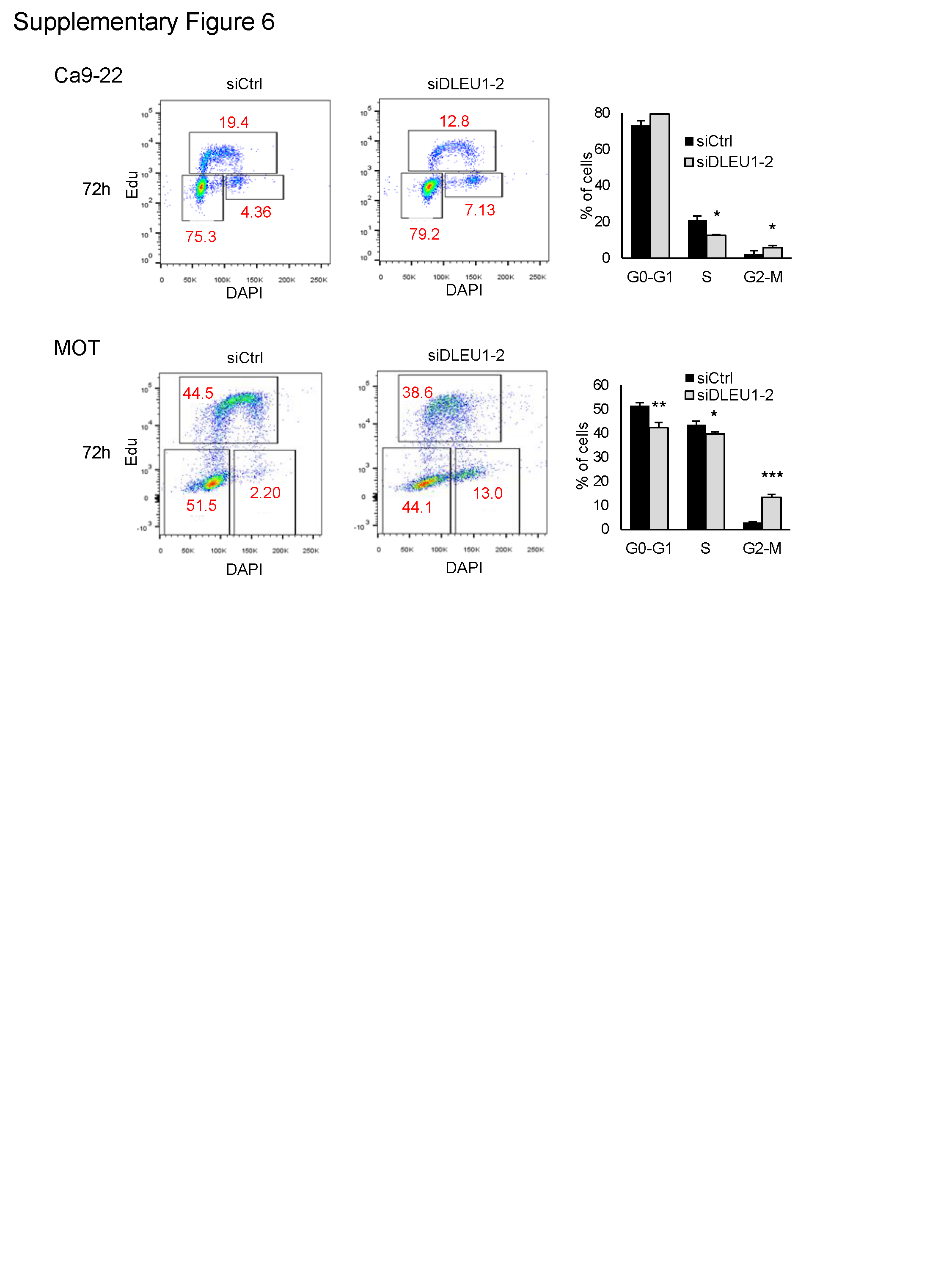


**Supplementary Figure 6**. Effects of DLEU1 knockdown on cell cycle in OSCC cells. Ca9-22 (upper) or MOT (lower) cells were transfected with siRNA targeting DLEU1 or control siRNA (siCtrl) and incubated for 72h, after which cell cycle was assessed. Representative results are shown on the left. Summarized results of 3 replications are on the right; error bars represent SDs. **P* < 0.05, ***P* < 0.01, ****P* < 0.001.


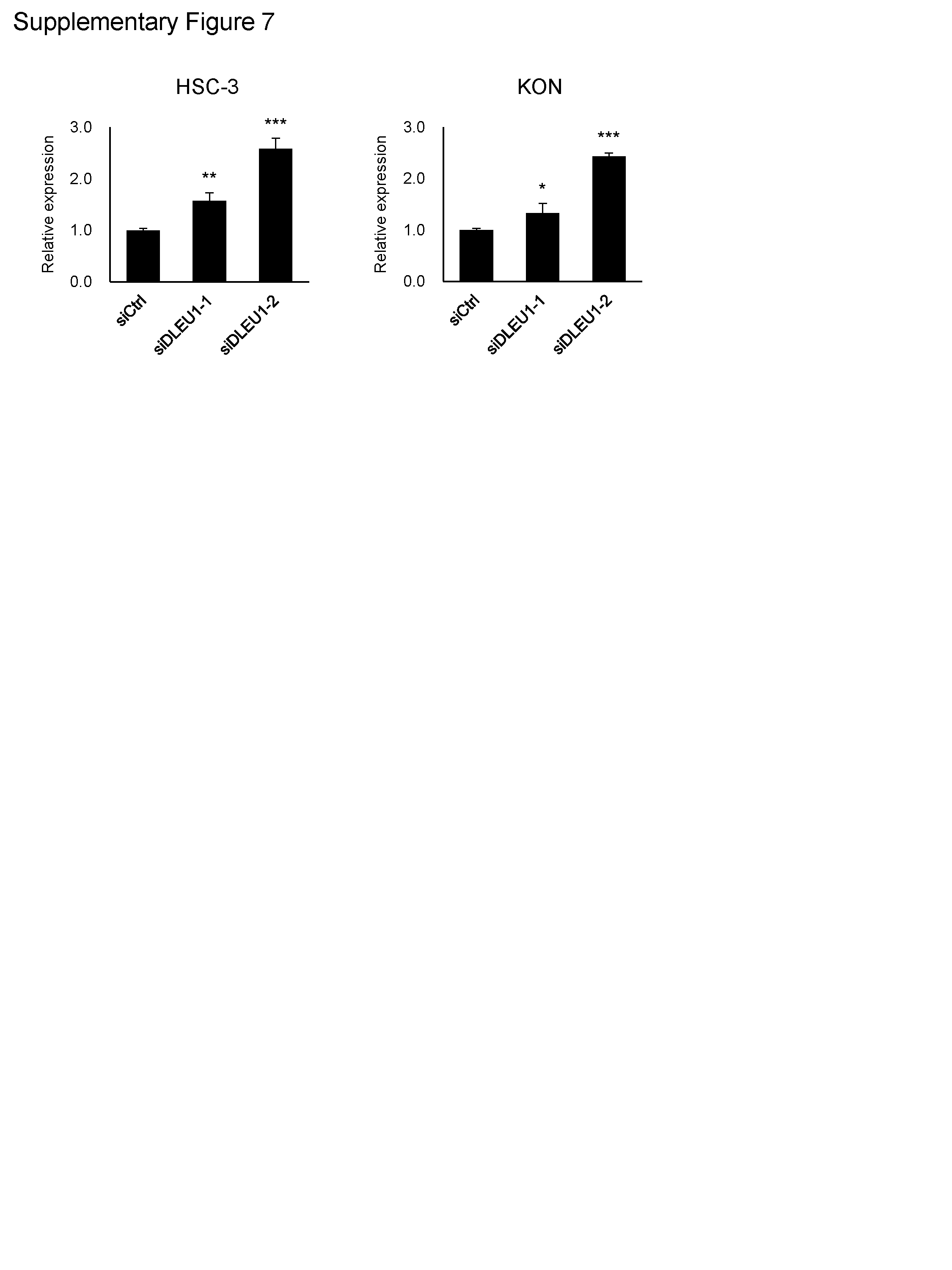


**Supplementary Figure 7**. DLEU1 knockdown induces WEE1 expression in OSCC cells. HSC-3 (left) or KON (right) cells were transfected with siRNAs targeting DLEU1 or control siRNA (siCtrl) and incubated for 72 h, after which expression of WEE1 was assessed using qRT-PCR. Shown are means of 3 replications; error bars represent SDs. **P* < 0.05, ***P* < 0.01, ****P* < 0.001.


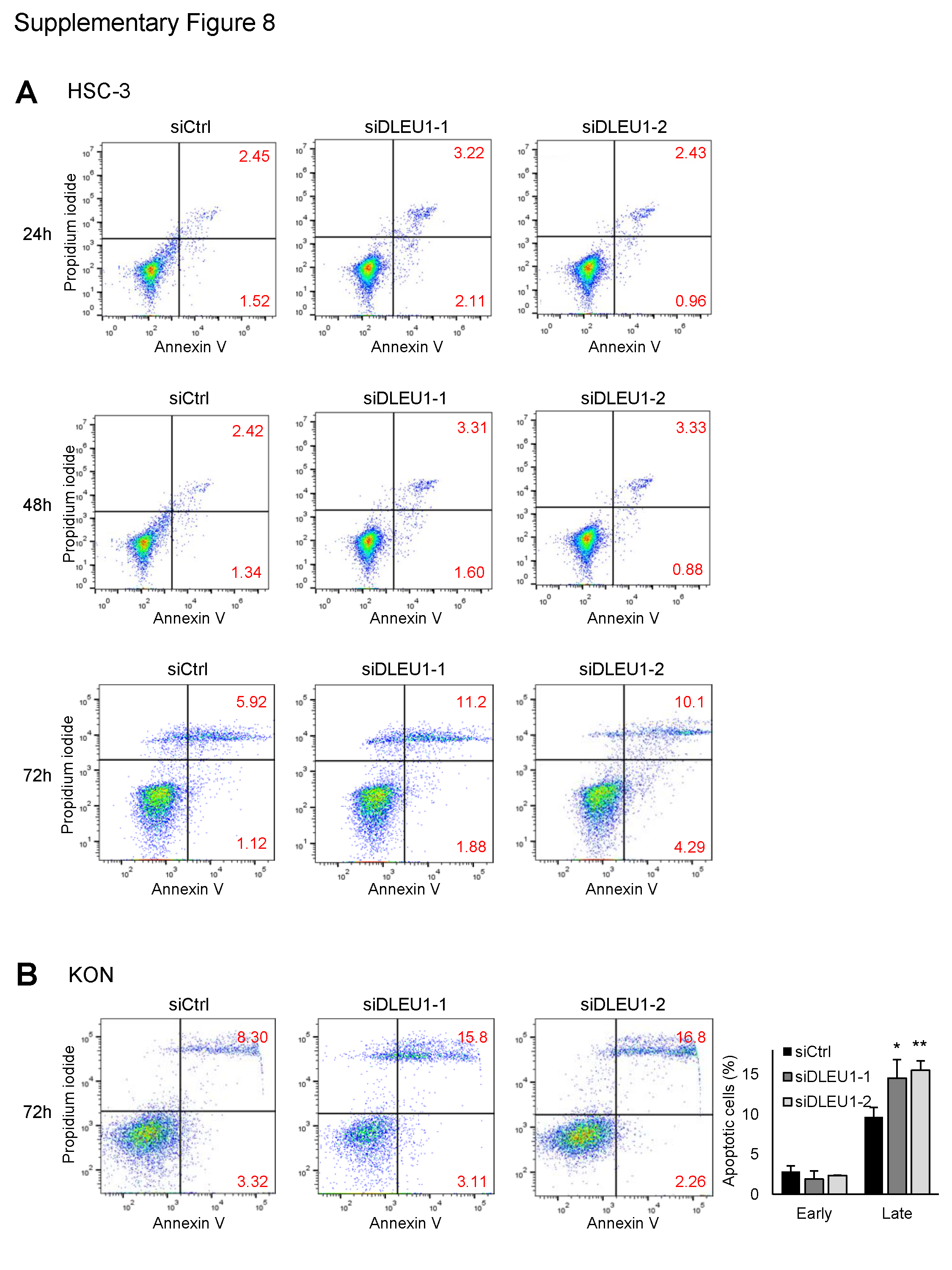


**Supplementary Figure 8**. DLEU1 knockdown induces apoptosis in OSCC cells. (A) Representative results of apoptosis analyses of HSC-3 cells transfected with siRNAs targeting DLEU1 or control siRNA (siCtrl). Cells were transfected with the indicated siRNAs, after which apoptosis was assessed at the indicated time points. Summarized results are shown in Figure 3. (B) Results of apoptosis analyses in KON cells transfected with the indicated siRNAs. Apoptosis was assessed 72 h after transfection. Representative results are shown on the left. Summarized results of 3 replications are on the right; error bars represent SDs. **P* < 0.05, ***P* < 0.01.


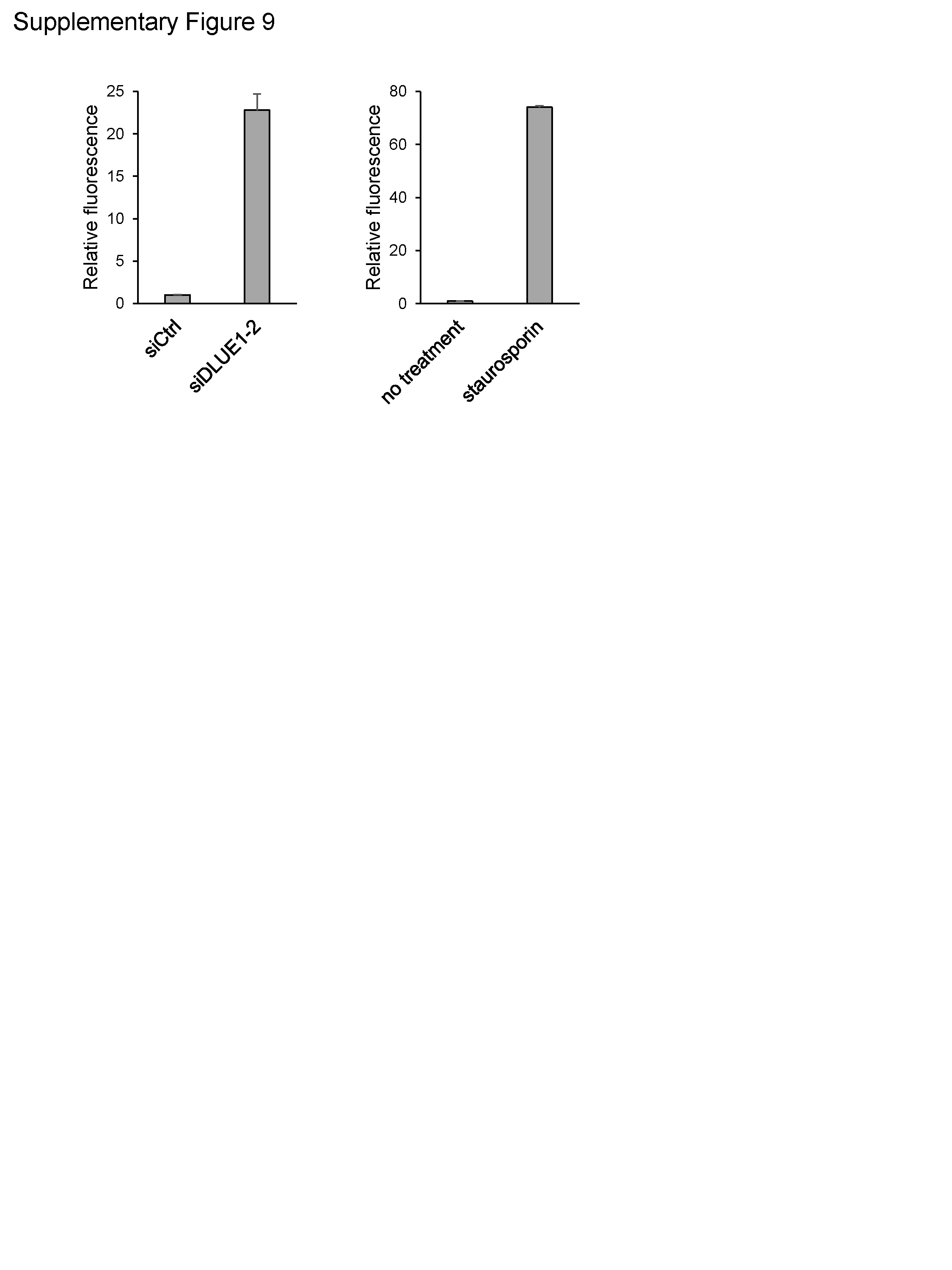


**Supplementary Figure 9**. DLEU1 knockdown enhances caspase-3 activity in OSCC cells. HSC-3 cells were transfected with the indicated siRNAs, and caspase-3 activities were assessed 72 h after transfection (left). HSC-3 cells treated with or without staurosporin (10 nM, 2 h) were used as positive and negative controls (right). Shown are means of 3 replications; error bars represent SDs.


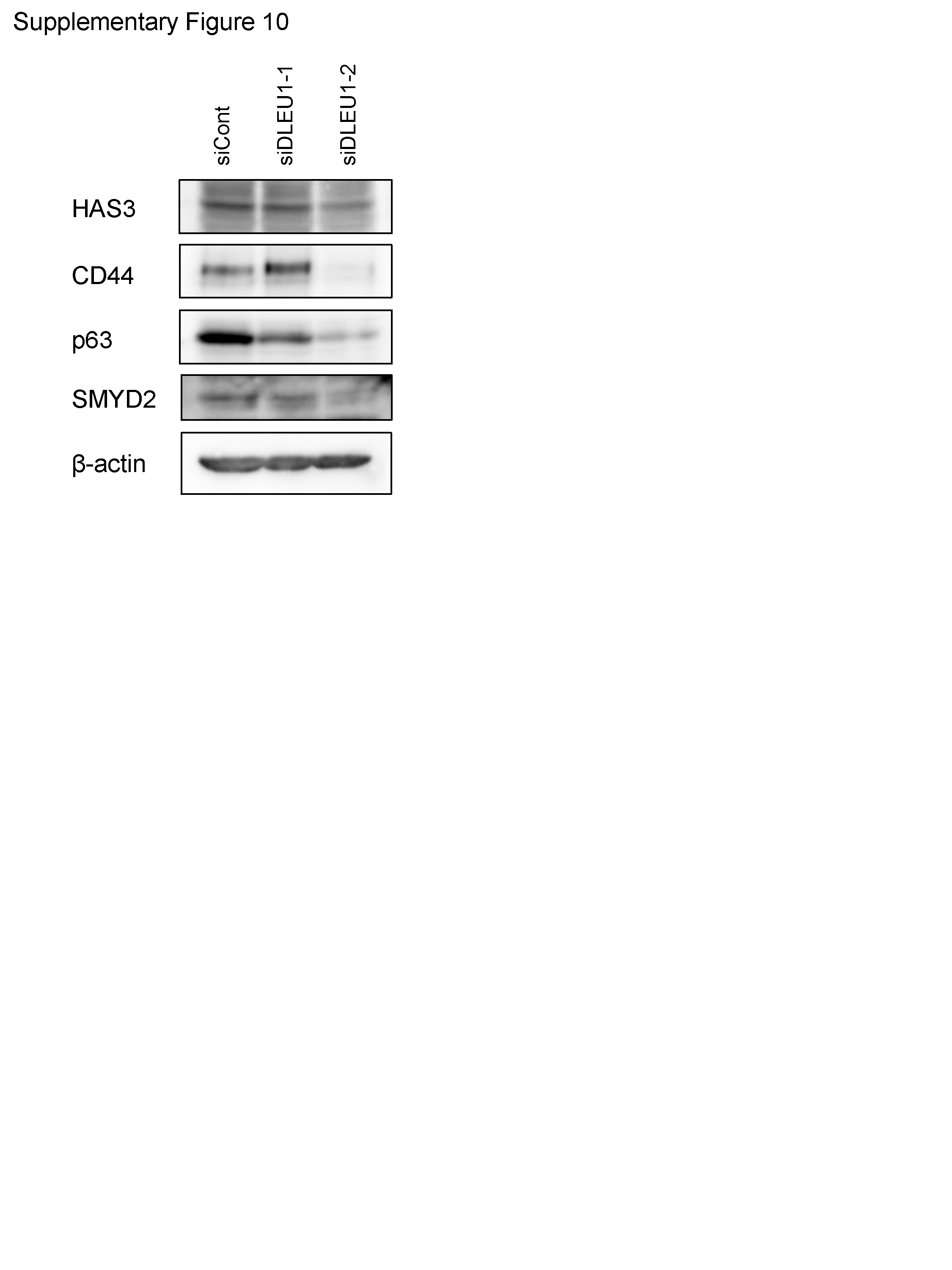


**Supplementary Figure 10**. Western blot analysis of HAS3, CD44, p63 and SMYD2 in OSCC cells after DLEU1 knockdown. HSC-3 cells were transfected with the indicated siRNAs, and cellular proteins were extracted 72 h after transfection. β-actin was used as a loading control.


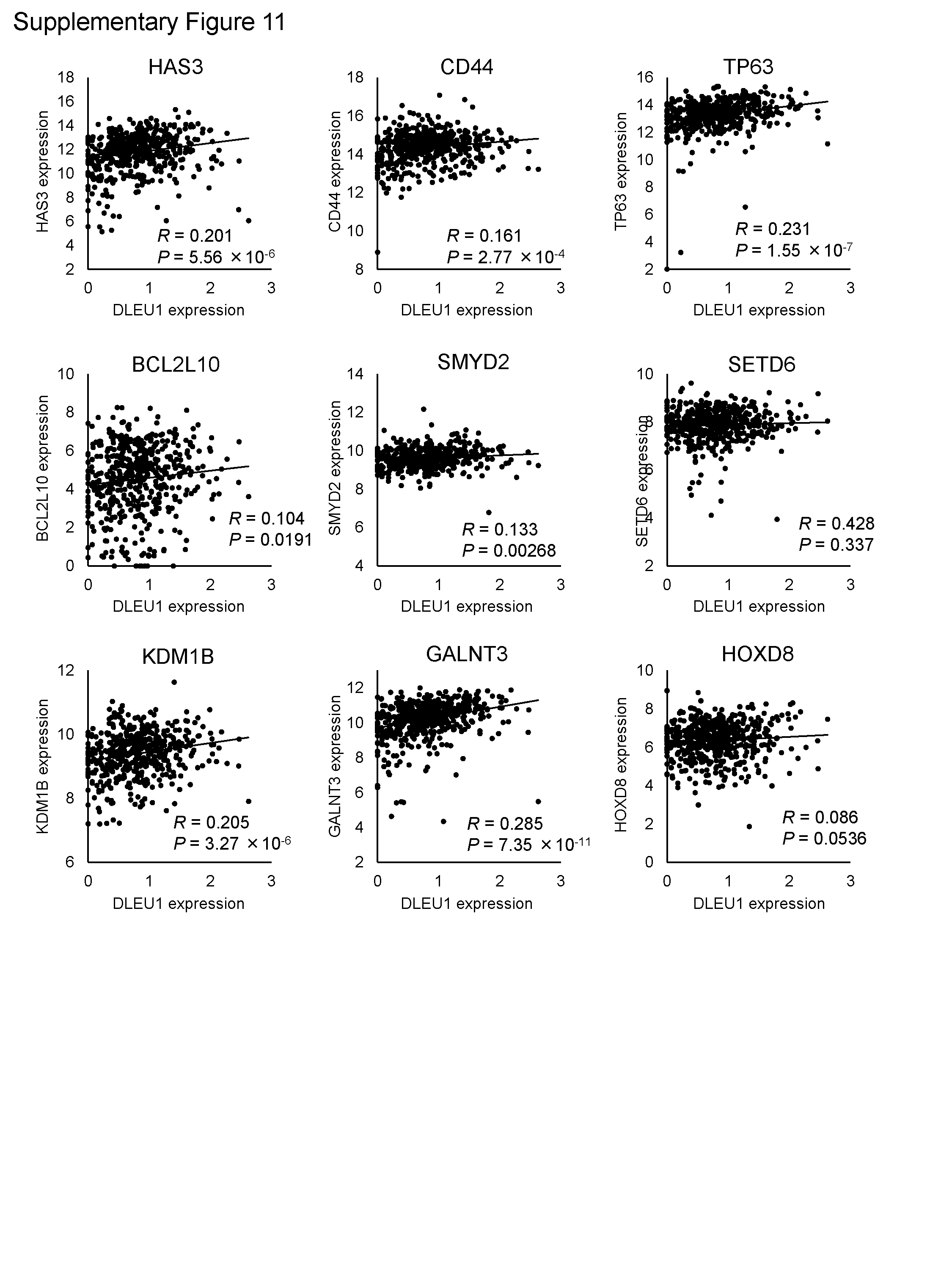


**Supplementary Figure 11**. Correlations between expression levels of DLUE1 and those of putative target genes in primary HNSCC in TCGA data sets. Pearson’s correlation coefficients and *P* values are shown.
